# Introduction for Proceedings volume of 16^th^ International Congress of Myriapodology

**DOI:** 10.3897/zookeys.510.10176

**Published:** 2015-06-30

**Authors:** Karel Tajovský, Ivan H. Tuf

**Affiliations:** 1 Institute of Soil Biology, Biology Centre CAS, Na Sádkách 7,České Budějovice, Czech Republic; 2 Department of Ecology and Environmental Sciences, Faculty of Science, Palacký University, Šlechtitelů 27, Olomouc, Czech Republic

The papers in this special issue of ZooKeys were presented at the 16th International Congress of Myriapodology (16ICM) held in Olomouc, the Czech Republic on July 20–25, 2014. International Congresses of Myriapodology are scientific meetings of scientists, students and enthusiastic amateurs with specific interest in millipedes, centipedes, symphylans and pauropods, as well as velvet worms. Those are organised under the auspices of the Centre International de Myriapodologie (http://www.myriapodology.org). To date, these congresses have a 46 year tradition and have taken place in 13 countries:

1ICM: 1968 – Paris, France

2ICM: 1972 – Manchester, UK

3ICM: 1975 – Hamburg, Germany

4ICM: 1978 – Gargnano, Italy

5ICM: 1981 – Radford, USA

6ICM: 1984 – Amsterdam, The Netherlands

7ICM: 1987 – Vittorio Veneto, Italy

8ICM: 1990 – Innsbruck, Austria

9ICM: 1993 – Paris, France

10ICM: 1996 – Copenhagen, Denmark

11ICM: 1999 – Bialowieza, Poland

12ICM: 2002 – Mtunzini, South Africa

13ICM: 2005 – Bergen, Norway

14ICM: 2008 – Görlitz, Germany

15ICM: 2011 – Brisbane, Australia

16ICM: 2014 – Olomouc, Czech Republic

**Figure 1.**
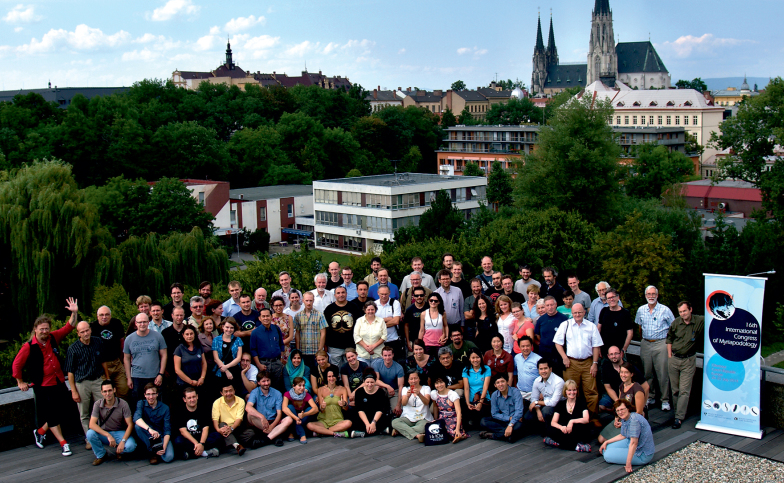
Collective photo of 16ICM participants.

**Figure 2.**
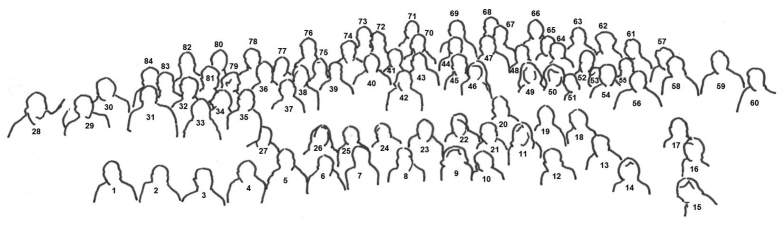
Legend of Fig. 1. **1** Boyan Vagalinski **2** Oliver Macek **3** Lucio Bonato **4** Hsueh-Wen Chang **5** Norman Lindner **6** Laura Del Latte **7** Irina Semenyuk **8** Michaela Bodner **9** Fieng-Lan Sun **10** Chao-chun Chen **11** Piyatida Pimvichai **12** Warut Siriwut **13** Natdanai Likhitrakarn **14** Małgorzata Leśniewska **15** Jolanta Wytwer **16** Michaela Kratochvílová **17** Christian Kronmüller **18** Cuong Huynh **19** Nattarin Wongthamwanich **20** João Paulo Pena-Barbosa **21** Julian Bueno-Villegas **22** Varpu Vahtera **23** Peter Decker **24** Barbara Jäschke **25** Julia Nefedieva **26** Roghaieh Zarei **27** Leszek Jendryszik **28** Ivan H. Tuf **29** Karel Tajovský **30** Sergei Ilyich Golovatch **31** Günther Raspotnig **32** Hans S. Reip **33** Darina Bachvarova **34** Elena Valentinovna Mikhaljova **35** Michalina Kszuk-Jendrysik **36** Pavel Stoev **37** Somsak Panha **38** Nesrine Akkari **39** Pavel Nefediev **40** Jean-Jacques Geoffroy **41** Giuseppe Fusco **42** Karin Voigtländer **43** Jean-Francois David **44** Bjarne Meidell **45** Amazonas Chagas-Jr. **46** Manoela Karam Gemael **47** Carsten H. G. Müller **48** Ivan Kos **49** Blanka Ravnjak **50** Maja Kastelic **51** Branka Vode **52** Megan Short **53** Hilke Ruhberg **54** Pavel Kocourek **55** Timotej Mock **56** Grzegorz Antoni Kania **57** John Lewis **58** Greg Edgecombe **59** Joseph Hannibal **60** Vladimír Šustr **61** Michal Rendoš **62** Andrej Mock **63** László Dányi **64** Ansgar Poloczek **65** Stylianos Simasiakis **66** Andy Sombke **67** Eivind Andreas Baste Undheim **68** Pavel Saska **69** Aleksandr Evsiukov **70** Willi Xylander **71** Bruce A. Snyder **72** Jan Philip Oeyen **73** Petr Dolejš **74** Henrik Enghoff **75** Ana Komerički **76** Markus Koch **77** Jörg Rosenberg **78** Iurii Diachkov **79** Daniela Bartel **80** Nikolaus U. Szucsich **81** Zoltán Korsós **82** Thomas Wesener **83** David Bogyo **84** Per Djursvoll. Aleksandar Doichinov, Elisavet Georgopoulou, Eszter Lazányi, Gabriella Papastefanou and Irina Zenkova are missing.

16ICM was the first myriapodological congress to be held in the Czech Republic. The 84 participants and 18 accompanying persons came from 24 countries on five continents, making the 16th Congress a truly international and global meeting of myriapod specialists (Figs 1, 2).

The organisers of the 16ICM are very grateful to the Palacký University, Olomouc, which provided us a pleasant area for all scientific as well as social parts of the Congress. We also thank the Biology Centre CAS and partial support from several unnamed colleagues who allowed the participation of young students at this Congress.

Many thanks to Lyubomir Penev and the friendly staff at Pensoft Publishers for making the 16ICM papers freely available to a global online audience.

